# Clinical Mortality Review of COVID-19 Patients at Sukraraj Tropical and Infectious Disease Hospital, Nepal; A Retrospective Study

**DOI:** 10.3390/tropicalmed6030137

**Published:** 2021-07-19

**Authors:** Anup Bastola, Sanjay Shrestha, Richa Nepal, Kijan Maharjan, Bikesh Shrestha, Bimal Sharma Chalise, Pratistha Thapa, Pujan Balla, Alisha Sapkota, Priyanka Shah

**Affiliations:** 1 Department of Tropical Medicine, Sukraraj Tropical and Infectious Disease Hospital, Kathmandu 44600, Nepal; docanup11@gmail.com; 2 Department of Internal Medicine, Sukraraj Tropical and Infectious Disease Hospital, Kathmandu 44600, Nepal; nepaldeepika123@gmail.com (R.N.); kijan1069@gmail.com (K.M.); bkeshshrestha4@gmail.com (B.S.); bschalise@gmail.com (B.S.C.); 3 Department of Anesthesiology, Sukraraj Tropical and Infectious Disease Hospital, Kathmandu 44600, Nepal; prats.thapa@gmail.com (P.T.); pujanballa@gmail.com (P.B.); 4 Department of Internal Medicine, Nepal Medical College and Teaching Hospital, Kathmandu 44600, Nepal; alishasapkota@gmail.com (A.S.); shahpriyanka543@gmail.com (P.S.)

**Keywords:** COVID-19, mortality, Nepal

## Abstract

Coronavirus Disease 2019 (COVID-19) has challenged the health system worldwide, including the low and middle income countries like Nepal. In view of the rising number of infections and prediction of multiple waves of this disease, mortalities due to COVID-19 need to be critically analyzed so that every possible effort could be made to prevent COVID-19 related mortalities in future. Main aim of this research was to study about the mortalities due to COVID-19 at a tertiary level hospital, in Nepal. This was a retrospective, observational study that included all inpatients from Sukraraj Tropical and Infectious Disease Hospital, who were reverse transcriptase polymerase chain reaction positive for SARS-COV-2 and died during hospital stay from January 2020 till January 2021. Medical records of the patients were evaluated. Out of 860 total admissions in a year, there were 50 mortalities in the study center. Out of 50 mortalities, majority were males (76%) with male to female ratio of 3.17:1. Most were above 65 years of age (72%) and had two or more comorbidities (64%). The most common comorbidities among the patients who had died during hospital stay were hypertension (58%) followed by diabetes mellitus (50%) and chronic obstructive airway disease (24%). The median duration from the symptom onset to death was 18 days, ranged from the minimum of 2 days till maximum of 39 days. D-dimer was found to be >1 mg/L in 58% cases and ferritin was >500 ng/ml in 42% patients at presentation. A total of 42% patients had thrombocytopenia, 80% patients had lymphocytopenia and 60% had Neutrophil to Lymphocyte ratio >11.75 with the mean NLR of 18.38. Of total mortalities, 16% patients also showed microbiological evidence of secondary infection; Male gender, age more than 65 years, multiple comorbidities with lymphocytopenia, elevated Neutrophil lymphocyte ratio and elevated inflammatory markers were risk factors found in majority of mortalities in our study. These findings could be utilized for early triage and risk assessment in COVID-19 patients so that aggressive treatment strategies could be employed at the earliest to reduce mortalities due to COVID-19 in future.

## 1. Introduction

Cases of pneumonia with unknown cause emerged in Wuhan, China, in December 2019 [1]. Epidemic investigation and gene sequencing revealed that a novel corona virus was the etiologic agent. The virus was tentatively named 2019-nCoV but officially named severe acute respiratory syndrome corona virus 2 (SARS-CoV-2) later by the Coronavirus Study Group of the International Committee on Taxonomy of Viruses and the disease caused by this virus was named as COVID-19 by the World Health Organization [2,3]. Globally, as of 7 July 2021, there have been 184,324,026 confirmed cases of COVID-19, including 3,992,680 deaths, reported to WHO [4].

It has been one year since Nepal reported its first COVID-19 patient. Sukraraj Tropical and Infectious Disease Hospital (STIDH), the only central government infectious disease hospital of Nepal, was the first to diagnose and treat the first-ever patient infected with COVID-19 in the country [5]. As of 7 July 2021, there have been 650,162 confirmed cases of COVID-19, including 9291 deaths accounting 1.42% of cases, reported to Ministry of Health and Population, Nepal [6]. 

The demographics of inpatient mortalities in published studies show association with different factors, such as age >60 years, male gender and multiple co-morbidities [7,8]. However, mortality data in Nepalese context is lacking. This study describes the demographic characteristics of COVID-19 mortalities in STIDH and observation of risk factors that contributed to mortality. As a central tropical and infectious disease hospital in Nepal, experiences of mortality at STIDH during this pandemic could help describe situational awareness and guide interventional strategies and responses at community and national levels, especially for low and middle income countries like Nepal.

## 2. Materials and Methods

### 2.1. Study Design and Participants

This was a retrospective, observational study that included all inpatients from Sukraraj Tropical and Infectious Disease Hospital, who were reverse transcriptase polymerase chain reaction positive for SARS-COV-2 and died during the study period from January 2020 to January 2021. 

Out of total 860 COVID-19 confirmed cases (reverse transcriptase polymerase chain reaction positive for SARS-COV-2) admitted at STIDH during the study duration, there were 50 mortalities, which were included in the study. 

COVID-19 cases were categorized as asymptomatic or pre-symptomatic, mild, moderate, severe and critical and were defined as follows [9]:

- Asymptomatic or pre-symptomatic infection: Individuals who test positive for SARS-CoV-2 using a virologic test (i.e., a nucleic acid amplification test or an antigen test) but who have no symptoms that are consistent with COVID-19.
- Mild illness: Individuals who have any of the various signs and symptoms of COVID-19 (e.g., fever, cough, sore throat, malaise, headache, muscle pain, nausea, vomiting, diarrhea, loss of taste and smell) but who do not have shortness of breath, dyspnea, or abnormal chest imaging.
- Moderate illness: Individuals who show evidence of lower respiratory disease during clinical assessment or imaging and who have peripheral capillary saturation of oxygen (SpO2) ≥ 94% on room air at sea level.
- Severe illness: Individuals who have SpO2 *<* 94% on room air at sea level, a ratio of arterial partial pressure of oxygen to fraction of inspired oxygen (PaO2/FiO2) < 300, respiratory frequency >30 breaths/min, or lung infiltrates >50%.
- Critical illness: Individuals who have respiratory failure, septic shock and/or multiple organ dysfunctions.

The study was approved by the Ethical Review Board of Nepal Health Research Council prior to start of data collection (Reference number 169/2021P, date of approval—21 March 2021) and permission was taken from the hospital director to include medical records of the inpatients of STIDH. Since this was a retrospective study and the data were analyzed anonymously, the requirement for taking consent was waived off by the ethical review board.

### 2.2. Ethics and Data Collection

Demographic, clinical, laboratory, treatment and outcome data were extracted from medical records using a data collection form. Two experienced clinicians reviewed and abstracted the data. Data were entered into a computerized database and cross-checked.

### 2.3. Statistical Analysis 

Statistical analyses were done using IBM Statistical Packages for Social Sciences (SPSS), version 20.0 (IBM Corp., IBM SPSS Statistics for Windows, Armonk, NY, USA). Continuous variables were directly expressed as median and interquartile range (IQR) values. Categorical variables were expressed as numbers and percentages (%). Pearson Chi-Square test and Fisher’s exact test were used as appropriate, based on the distribution and *p*-values were tabulated with a level of significance set at <0.05.

## 3. Results

This section may be divided by subheadings. It should provide a concise and precise description of the experimental results, their interpretation, as well as the experimental conclusions that can be drawn.

### 3.1. Clinical Characteristics of Total Patients Admitted in STIDH

**Figure 1:**
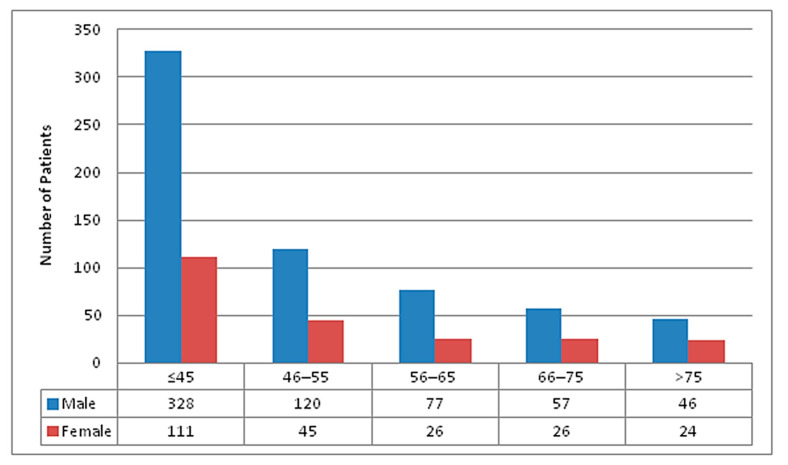
Age and Gender wise distribution of total COVID-19 patients admitted in STIDH (N = 860).

Out of total 860 COVID-19 patients admitted over the period of one year, 628 (73%) were males and 232 (27%) were females with the male to female ratio of 2.7:1. A total of 439 (51%) patients were ≤45 years, 165 (19.2%) were from age group 46–55 years, 103 (12.0%) were from age group 56–65 years, 83 (9.7%) were from age group 66–75 years and 70 (8.1%) patients were more than 75 years of age. Age and gender wise distribution of total admitted patients has been represented in Figure 1.

**Figure 2:**
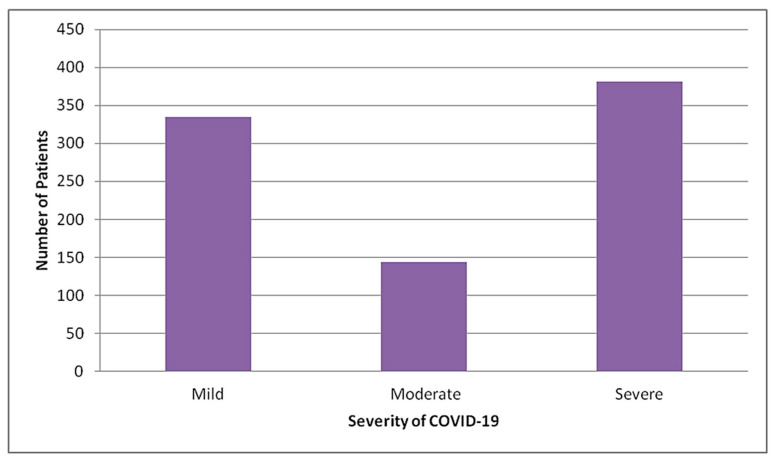
Distribution of total patients admitted in STIDH by severity of COVID-19 (N = 860).

Out of total admitted patients, the majority (44.3%) had severe COVID-19, 16.7% had moderate COVID-19 and remaining 39% had mild COVID-19 (Figure 2).

**Figure 3:**
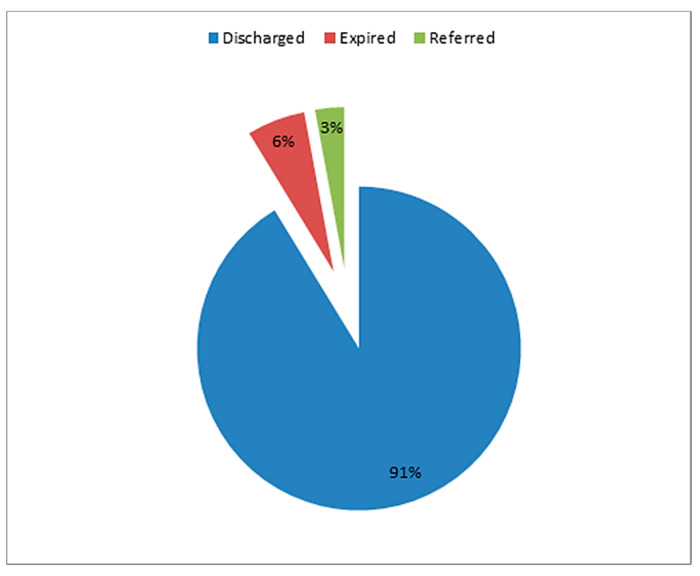
Outcome of total patients admitted in STIDH (N = 860).

Among 860 patients admitted in our facility, 50 (5.8%) patients had died whereas 25 (2.9%) patients had to be referred to other center either due to the need of mechanical ventilation which could not be made available to few patients at our center during the peak of pandemic or due to the need of multispecialty care which included dialysis or concomitant surgical care (Figure 3).

### 3.2. Clinical Characteristics of the Mortalities in STIDH

#### 3.2.1. Baseline Characteristics

**Figure 4:**
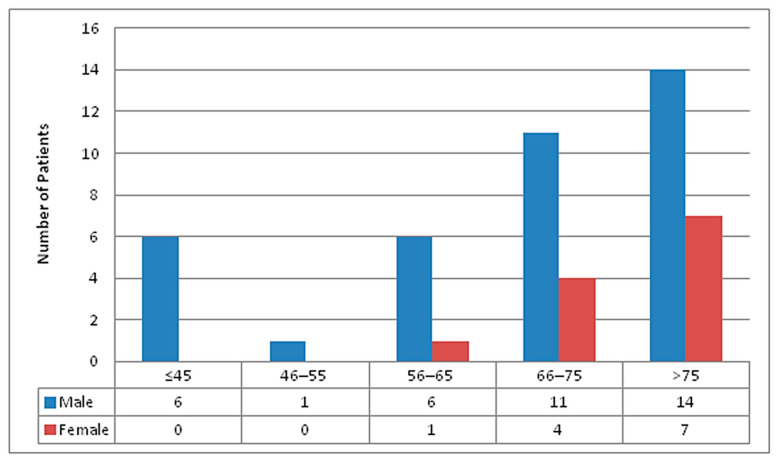
Age and gender wise distribution of mortalities in STIDH (n = 50).

Among 50 deaths recorded, 38 (76%) were males and 12 (24%) were females, with male to female ratio of 3.17:1. The median age was 72.5 years, ranging from 36 to 95 years. Most (21/50, 42%) patients were of the age group ≥76 years, followed by 66–75 years (15/50, 30%). Distribution of total mortalities by gender and age groups are shown in Figure 4. 

**Figure 5:**
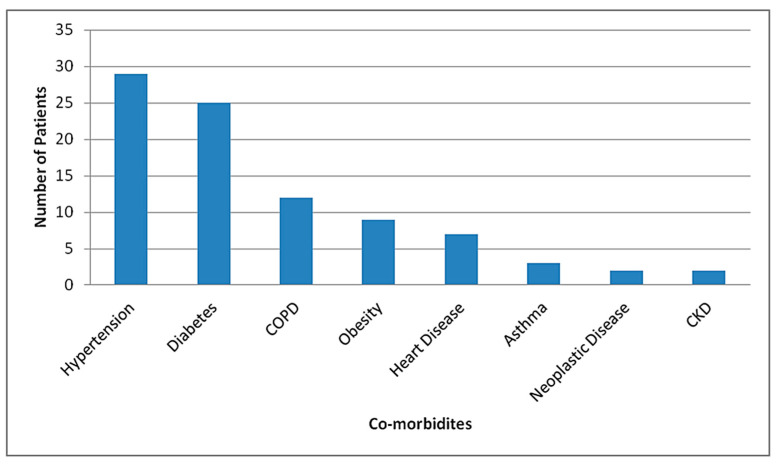
Distribution of co-morbidities among mortalities in STIDH (n = 50).

41 patients (82%) who died of COVID-19 had underlying disease, the most common of which was hypertension (29/50, 58%), followed by diabetes (25/50, 50%), chronic obstructive pulmonary disease (12/50, 24%), obesity (9/50, 18%), heart disease (7/50, 14%), asthma (3/50, 6%), neoplastic disease (2/50, 4%) (One had carcinoma of gall bladder and the other had multiple myeloma) and chronic kidney disease (1/50, 2%) (Figure 5). A total of 33 (66%) patients who died during hospital stay had multiple (two or more than two) co-morbidities in our study.

Multiple comorbidities (2 or more) were likely to be present in patients with increasing age with statistically significant association (Table 1).

The presence of multiple comorbidities, however, did not seem to have a significant association with sex (Table 2).

#### 3.2.2. Clinical Presentation 

**Figure 6:**
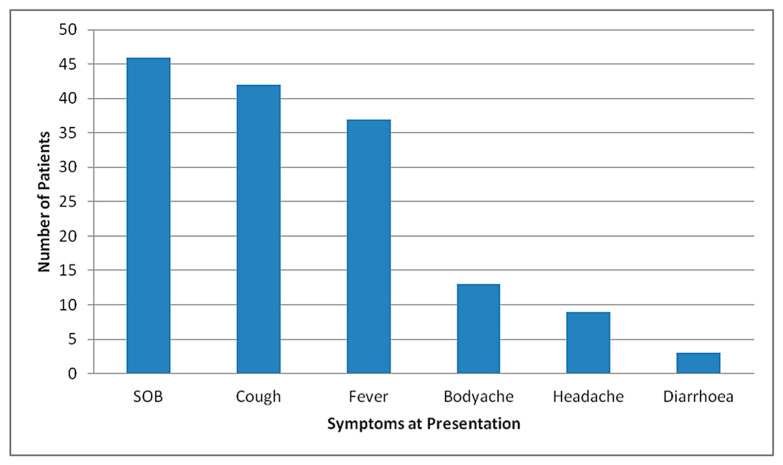
Distribution of symptoms at presentation among mortalities at STIDH (n = 50).

The median duration of symptoms prior to admission was 5 days, ranging from 1–20 days. The median duration of hospital stay was 10.5 days, ranging from 1–35 days. The median duration from the first symptom to death was 18 days, ranging from 2–39 days. For male patients, the median duration from the first symptom to death was 18.5 days and for female patients was 13.5 days. Most of the patients presented as severe disease (47/50, 94%), rest (3/50, 6%) presented as moderate disease and later progressed. Shortness of breath was the most common reported symptom among patients who eventually died (46/50, 92%), followed by cough (42/50, 84%), fever (37/50, 74%), body ache (13/37, 26%), headache (9/50, 18%) and diarrhea (2/50, 4%), as represented in Figure 6.

#### 3.2.3. Biomarkers and Hematological Variables

**Figure 7:**
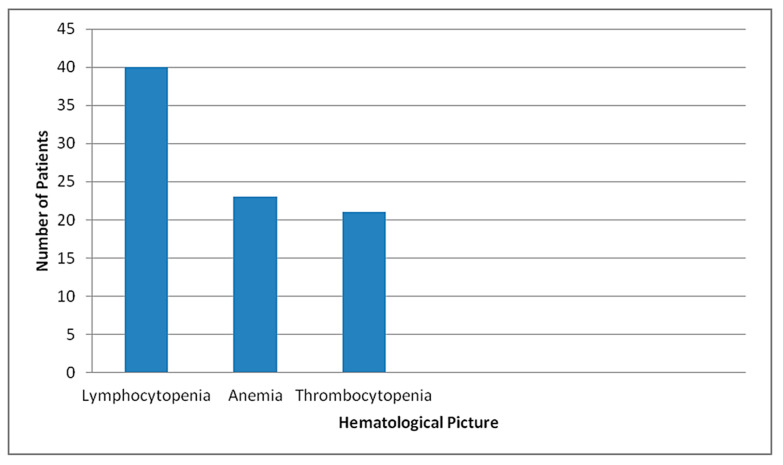
Distribution by hematological picture of the mortalities in STIDH (n = 50).

C-reactive protein was positive in 42/50 (84%) cases. D-dimer was found to be >1 mg/L in 29/50 (58%) cases and ferritin was >500 ng/ml in 21/50 (42%) patients at presentation. A total of 14 patients had repeated D-dimer measurements, which showed increase in the level of D- dimer. A total of 23/50 (46%) patients were anemic with the mean hemoglobin level being 12.85 g/dL, ranging from 9.6 to 18.4 g/dL. A total of 21/50 (42%) patients had thrombocytopenia, while 4/50 (8%) had thrombocytosis at presentation. A total of 25/50 (50%) had normal platelet count. Of 21 patients with thrombocytopenia, 10 had moderate and 11 had mild thrombocytopenia. A total of 40/50 (80%) patients had lymphocytopenia (absolute lymphocyte count <1000 cells/µL) at presentation (Figure 7). Neutrophil to lymphocyte ratio was >11.75 in 30/50 (60%) patients, with the mean NLR being 18.38, ranged from 1.52 to 98. A total of 10/50 (20%) patients had renal impairment and 4/50 (8%) had transaminitis at presentation. A total of 29/50 (58%) developed acute kidney injury during hospital stay, while 10/50 (20%) developed hepatic impairment. 

#### 3.2.4. Microbiology

A total of 8/50 (16%) patients showed microbiological evidence of secondary infection, 6/50 (12%) patients had sputum culture positive for organisms and 2/50(4%) had urine culture positivity. *Klebsiella pneumoniae* was the predominant organism in sputum culture (5/6, 83.3%) followed by *Escherichia coli* (1/6, 16.7%). A total of 2/50 (4%) patients had urine culture positive for *E. coli*.

#### 3.2.5. Treatment

**Figure 8:**
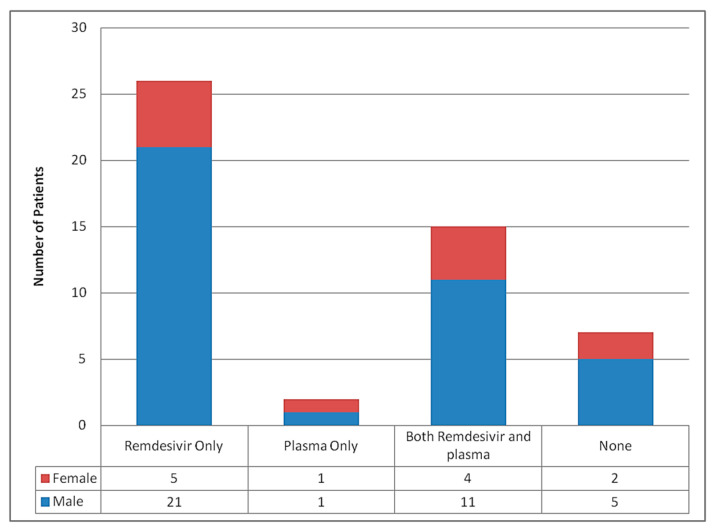
Treatment according to the gender of patients who died.

The antiviral drug remdesivir was used in 41/50 (82%) patients, while convalescent plasma transfusions were used in 17/50 (34%) patients (Figure 8). A total of 6/41 (14.6%) patients received remdesivir for 10 days, 27/41 (65.9%) received for 5 days. One patient received for 4 days, while 2 received for 3, 2 for 2 days and 2 for 1 day. Some died before completion of therapy and in some patients; the drug was stopped prematurely when they developed significant hepatic or renal impairment limiting the use of drug. A total of 31/50(62%) patients received therapeutic anticoagulation with low molecular weight heparin (LMWH), whereas 19/50 (38%) patients had only received prophylactic anticoagulation with heparin. The highest level of oxygen delivery device used in most patients was non-invasive ventilation in 40/50 (80%) patients. Invasive ventilation was only used in 7/50 (14%) patients, followed by the use of reservoir mask in 2/50 (4%) patients and High Flow Nasal Cannula (HFNC) in 1/50 (2%) patient. The reason for inability to escalate respiratory support to invasive mechanical ventilation was due to the do not resuscitate status of patients as per the wishes of patient’s party after knowing about grave prognosis and also due to logistic issues at our center.

#### 3.2.6. Cause of Death

A total of 40/50 (80%) deaths were due to type 1 respiratory failure, while 7/50 (14%) died of septic shock and 3/50 (6%) died of multiple organ dysfunction syndrome. The contributory cause was AKI in 20/50 (40%) patients and sepsis in 5/50 (10%) patients.

## 4. Discussion

This study represents a review of the COVID-19 deaths at Sukraraj Tropical and Infectious Disease Hospital during the first wave of coronavirus pandemic in Nepal from early 2020 till early 2021. Patients with underlying diseases and age over 65 years made up the majority of deaths, similar to demographics of COVID-19 deaths from other studies [10,11,12,13]. Du et al. performed a single center prospective cohort study to investigate the possible risk factors associated with the poorest clinical outcome (dying from COVID-19 pneumonia) and reported that age ≥65 years to be one of the predictors for mortality in COVID-19 [10]. The current study also suggested that old age was associated with deaths in patients with COVID-19. Age as a determinant for prognosis could be due to poor health and associated comorbidities. This signifies the need for aggressive monitoring and intensive treatment strategies to be employed at the earliest to limit deaths in older age population. Further, prophylactic treatments in anticipation of the disease in the elderly could involve natural repetitive stimulations of the heat shock response in the whole body through controlled intense physical exercise, sauna therapies and the regular maintenance of calorie-restricted diets containing minimal amounts of saturated lipids and cholesterol [14].

Recent studies revealed lymphocytopenia as an important characteristic of SARS-CoV-2 infection, especially in critically ill and deceased patients [11,12]. Huang et al. and Wang et al. showed an association between lymphocytopenia and need of intensive care unit [1,13]. Similarly, Wu et al. showed an association between lymphocytopenia and acute respiratory distress syndrome (ARDS) development [15]. In Singapore, Fan et al. found that patients requiring ICU support had significantly lower lymphocyte levels at baseline [16]. Similar to these studies, our study also showed that the majority (80%) of patients who died had lymphocytopenia at presentation. Thus, lymphocytopenia can be taken as one of the severity markers of COVID-19 and we need to be vigilant in patients presenting with lymphocytopenia.

A meta-analysis of nine studies suggested that thrombocytopenia is significantly associated with the severity of COVID-19 disease; a more sizeable drop in platelet counts was noted especially in non-survivors [17]. In our study, significant proportion (42%) of cases that died had thrombocytopenia at presentation. A systematic review and meta-analysis by Li et al. concluded that NLR has significant predictive value for disease severity and mortality in patients with COVID-19 infection [18]. Similarly, Liu Y et al. showed that NLR is an independent risk factor of the in-hospital mortality for COVID-19 patients [19]. A retrospective cross-sectional study done by Yan et al. showed that NLR more than 11.75 was significantly correlated with all-cause in-hospital mortality [20]. In our study, the majority of patients who died presented with NLR > 11.75. NLR evaluation can help clinicians identify potentially severe cases early, conduct early triage and initiate aggressive management at the earliest, which may reduce the overall mortality of COVID-19.

A retrospective, multi-center cohort study from Jinyintan Hospital and Wuhan Pulmonary Hospital (Wuhan, China) showed that older age, D-dimer levels greater than 1 μg/mL and higher SOFA score on admission were associated with higher odds of in-hospital death [21]. A meta-analysis done by Huang et al. also suggested that elevated serum C-reactive protein, procalcitonin, D-dimer and ferritin were associated with poor outcome in COVID-19. It showed that a D-dimer >0.5 mg/dL had 58% sensitivity and 69% specificity for severe disease [22]. In a retrospective study by Tang et al. encompassing data from 183 consecutive patients with COVID-19, non-survivors had significantly higher D-dimer (*p* < 0.05), fibrin degradation product (FDP) levels (*p* < 0.05), prolonged PT (*p* < 0.05) and APTT (*p* < 0.05) compared with survivors at initial evaluation [23]. In a multicenter retrospective cohort study from China by Wu et al, increased D-dimer levels (>1 μg/mL) were significantly associated with in-hospital death in the multivariable analysis [15]. Similarly, the same study also showed that higher serum ferritin was associated with ARDS development. Zhou et al. also supported an association between higher serum ferritin levels and death [21]. D-dimer was high (>1.0 mg/L) in 58% of our patients and high ferritin levels (>500 ng/ml) were present in 42% patients who had died at our center during the study duration. Follow-up D-dimer estimation in 14 patients showed increased levels. Thus, we conclude that the D-dimer dynamics can reflect the severity of disease and their increased levels are associated with adverse outcomes among patients with COVID-19. 

Our study has some limitations. Since this was a single centered study, the results cannot be generalized. Secondly, being a retrospective study, laboratory tests of all the patients were not available, including quantitative CRP measurements, D-dimer and serum ferritin levels. Therefore, their role might have been underestimated in predicting the in-hospital deaths. Thirdly, some patients were transferred late in their illness to our hospital. Lack of early interventions and inadequate adherence to standard supportive therapy, might have also contributed to the poor clinical outcome in some patients. Fourthly, a significant number of critical patients were referred to centers due to multiple reasons. Follow up and outcome of those patients were not included in this study. Furthermore, data related to thromboembolism, which is a significant cause of COVID-19 related death, could not be investigated due to logistic limitation. 

## 5. Conclusions

In summary, most of the COVID-19 deaths in our study were of older age, male gender and had multiple comorbidities. Hematologically, lymphocytopenia and increased neutrophil to lymphocyte ratio were found in the COVID-19 mortalities. Similarly, elevated D-dimer and ferritin at admission were risk factors for death of adult patients with COVID-19. Evaluation of these factors during admission might help us for risk assessment and early triage of potential severe COVID-19 cases so that every possible effort can be made for the prompt management of COVID-19 and avoidance of deaths.

## Figures and Tables

**Table 1:** Age wise distribution of comorbidities among mortalities in STIDH (n = 50).

Age Group	Multiple (2 or More) Comorbidities		Total	*p*-Value
	Absent (n)	Present (n)		
≤45	5	1	6	0.001
46–55	1	0	1	
56–65	5	2	7	
66–75	4	11	15	
>75	2	19	21	
Total	17	33	50	

**Table 2:** Sex wise distribution of multiple comorbidities among mortalities in STIDH (n = 50).

Sex	Multiple (2 or More) Comorbidities		Total	*p*-Value
	Absent (n)	Present (n)		
Female	3	9	12	0.510
Male	14	24	38	
Total	17	33	50	

## Data Availability

Data will be made available from the corresponding author on request.

## References

[1] Huang C., Wang Y., Li X., Ren L., Zhao J., Hu Y., Zhang L., Fan G., Xu J., Gu X. (2020). Clinical features of patients infected with 2019 novel coronavirus in Wuhan, China. Lancet.

[2] Lu R., Zhao X., Li J., Niu P., Yang B., Wu H., Wang W., Song H., Huang B., Zhu N. (2020). Genomic characterisation and epidemiology of 2019 novel coronavirus: Implications for virus origins and receptor binding. Lancet.

[3] Zhu N., Zhang D., Wang W., Li X., Yang B., Song J., Zhao X., Huang B., Shi W., Lu R. (2020). A novel coronavirus from patients with pneumonia in China, 2019. N. Engl. J. Med..

[4] WHO Coronavirus (COVID-19) Dashboard|WHO Coronavirus (COVID-19) Dashboard with Vaccination Data. https://covid19.who.int/.

[5] Bastola A., Sah R., Rodriguez-Morales A.J., Lal B.K., Jha R., Ojha H.C., Shrestha B., Chu D.K., Poon L.L., Costello A. (2020). The first 2019 novel coronavirus case in Nepal. Lancet Infect. Dis..

[6] CoVid19-Dashboard. https://covid19.mohp.gov.np/.

[7] Jarrett M.P., Schultz S.F., Lyall J.S., Wang J.J., Stier L., De Geronimo M., Nelson K.L. (2020). Clinical Mortality Review in a Large COVID-19 Cohort. medRxiv.

[8] Yancy C.W. (2020). COVID-19 and african americans. JAMA.

[9] National Institutes of Health COVID-19 Treatment Guidelines Panel. Coronavirus Disease 2019 (COVID-19) Treatment Guidelines. https://www.covid19treatmentguidelines.nih.gov/.

[10] Du R.-H., Liang L.-R., Yang C.-Q., Wang W., Cao T.-Z., Li M., Guo G.-Y., Du J., Zheng C.-L., Zhu Q. (2020). Predictors of mortality for patients with COVID-19 pneumonia caused by SARS-CoV-2: A prospective cohort study. Eur. Respir. J..

[11] Yang X., Yu Y., Xu J., Shu H., Liu H., Wu Y., Zhang L., Yu Z., Fang M., Yu T. (2020). Clinical course and outcomes of critically ill patients with SARS-CoV-2 pneumonia in Wuhan, China: A single-centered, retrospective, observational study. Lancet Respir. Med..

[12] Chen T., Wu D., Chen H., Yan W., Yang D., Chen G., Ma K., Xu D., Yu H., Wang H. (2020). Clinical characteristics of 113 deceased patients with coronavirus disease 2019: Retrospective study. BMJ.

[13] Wang D., Hu B., Hu C., Zhu F., Liu X., Zhang J., Wang B., Xiang H., Cheng Z., Xiong Y. (2020). Clinical characteristics of 138 hospitalized patients with 2019 novel Coronavirus—Infected pneumonia in Wuhan, China. JAMA.

[14] Guihur A., Rebeaud M.E., Fauvet B., Tiwari S., Weiss Y.G., Goloubinoff P. (2020). Moderate Fever Cycles as a Potential Mechanism to Protect the Respiratory System in COVID-19 Patients. Front. Med..

[15] Wu C., Chen X., Cai Y., Zhou X., Xu S., Huang H., Zhang L., Zhou X., Du C., Zhang Y. (2020). Risk factors associated with acute respiratory distress syndrome and death in patients with coronavirus disease 2019 pneumonia in Wuhan, China. JAMA Intern. Med..

[16] Fan B.E. (2020). Hematologic parameters in patients with COVID-19 infection: A reply. Am. J. Hematol..

[17] Lippi G., Plebani M., Henry B.M. (2020). Thrombocytopenia is associated with severe coronavirus disease 2019 (COVID-19) infections: A Meta—Analysis. Clin. Chim. Acta.

[18] Li X., Liu C., Mao Z., Xiao M., Wang L., Qi S., Zhou F. (2020). Predictive values of neutrophil-to-lymphocyte ratio on disease severity and mortality in COVID-19 patients: A systematic review and meta-analysis. Crit. Care.

[19] Liu Y., Du X., Chen J., Jin Y., Peng L., Wang H.H., Luo M., Chen L., Zhao Y. (2020). Neutrophil-to-lymphocyte ratio as an independent risk factor for mortality in hospitalized patients with COVID-19. J. Infect..

[20] Yan X., Li F., Wang X., Yan J., Zhu F., Tang S., Deng Y., Wang H., Chen R., Yu Z. (2020). Neutrophil to lymphocyte ratio as prognostic and predictive factor in patients with coronavirus disease 2019: A retrospective Cross—Sectional study. J. Med. Virol..

[21] Zhou F., Yu T., Du R., Fan G., Liu Y., Liu Z., Xiang J., Wang Y., Song B., Gu X. (2020). Clinical course and risk factors for mortality of adult inpatients with COVID-19 in Wuhan, China: A retrospective cohort study. Lancet.

[22] Huang I., Pranata R., Lim M.A., Oehadian A., Alisjahbana B. (2020). C-reactive protein, procalcitonin, D-dimer, and ferritin in severe coronavirus disease-2019: A meta-analysis. Ther. Adv. Respir. Dis..

[23] Tang N., Li D., Wang X., Sun Z. (2020). Abnormal coagulation parameters are associated with poor prognosis in patients with novel coronavirus pneumonia. J. Thromb. Haemost..

